# Magnitude of Glycemic Improvement in Patients with Type 2 Diabetes Treated with Basal Insulin: Subgroup Analyses from the MOBILE Study

**DOI:** 10.1089/dia.2021.0489

**Published:** 2022-05-10

**Authors:** Georgia Davis, Ryan Bailey, Peter Calhoun, David Price, Roy W. Beck

**Affiliations:** ^1^ Emory University School of Medicine, Department of Medicine, Division of Endocrinology, Metabolism, and Lipids, Atlanta, GA.; ^2^ Jaeb Center for Health Research, Tampa, Florida, USA.; ^3^ Dexcom, Inc., San Diego, California, USA.

**Keywords:** Continuous glucose monitoring, Basal insulin, Glycemic management, Time-in-range

## Abstract

**Objective:**

To determine if type 2 diabetes patients using basal insulin without prandial insulin with worse glycemic control at baseline would have the greatest benefit from using real-time continuous glucose monitoring (CGM).

**Methods:**

We conducted a post hoc analysis of the MOBILE Study, a multicenter trial examining the impact of CGM versus self-monitoring with a blood glucose meter (BGM) in patients with type 2 diabetes treated with basal insulin without prandial insulin. Participants were divided into subgroups based on baseline hemoglobin A1c (HbA1c) and baseline time-in-range 70–180 mg/dL (TIR). Change in TIR from baseline was calculated within each subgroup.

**Results:**

In subgroups based on baseline HbA1c, compared with the BGM group, the CGM group had 14% greater increase in TIR for participants with baseline HbA1c ≥8.5%, 14% greater increase for baseline HbA1c ≥9.0%, 22% greater increase for baseline HbA1c ≥9.5%, and 32% greater increase for baseline HbA1c ≥10.0% (*P*-value for interaction = 0.27). The time spent with glucose >250 mg/dL was significantly lower with CGM compared with BGM among participants with higher HbA1c values (*P* for interaction = 0.004). Results in subgroups based on baseline TIR paralleled the results in subgroups based on baseline HbA1c.

**Conclusion:**

While the benefit of CGM on TIR among patients with type 2 diabetes treated with basal insulin is apparent across the range of baseline glycemic control, the greatest impact of CGM is in those with the worst baseline glycemic control, particularly among those with HbA1c ≥10%.

Clinical Trial Registration number: NCT03566693.

## Introduction

The use of real-time continuous glucose monitoring (CGM) devices for diabetes management has been expanding rapidly during the past decade. Previous trials investigating the use of this technology for the management of both type 1 and type 2 diabetes have focused on those requiring intensive insulin therapy using multiple daily injections or continuous subcutaneous insulin infusion.^1–6^ With increasing access to CGM technology, there is interest in understanding the utility of CGM for improving glycemic control in patients not requiring intensive insulin therapy. The way CGM may influence glycemic control in patients not receiving intensive insulin therapy is multifaceted, with a potentially greater impact arising from improvements in glucose monitoring, medication adherence, lifestyle modifications, and health care provider interactions.^7–9^

There is limited evidence that CGM may improve glycemic control in patients with type 2 diabetes on variable treatment regimens,^7,8,10,11^ although it is not clear which patients may experience the most treatment benefit from ongoing CGM therapy.

Clinical trials evaluating diabetes treatment interventions have consistently shown that those with worse baseline glycemic control are more likely to experience a greater treatment response.^12–15^ This varying degree of efficacy also has been shown through analysis of results from prior CGM studies, with the largest improvement in hemoglobin A1c (HbA1c) observed in those with the highest values at baseline.^16–19^ However, the majority of previous CGM studies have enrolled patients on intensive insulin therapy, where improved glycemic control may be attributed to the ability to modulate prandial or corrective insulin delivery.^20^ There is emerging data suggesting that the use of CGM can improve glycemic control in patients with type 2 diabetes treated with less intensive insulin regimens or with noninsulin agents alone.^7,8^ However, it is unknown if a greater treatment response in those with worse baseline glycemic control persists in these populations without the use of short or rapid acting insulin.

The recent multicenter MOBILE clinical trial showed that sustained use of real-time CGM (CGM) in participants with type 2 diabetes treated with basal insulin in combination with other noninsulin agents in a primary care setting improved glycemic control during 8 months of follow-up.^21^ Both HbA1c and mean glucose values after 8 months were lower for participants using CGM compared to those using self-monitoring of blood glucose with a blood glucose meter (BGM).^21^ To further evaluate the magnitude of the effect of CGM on glycemic control among adults with poorly controlled type 2 diabetes treated with basal insulin, we examined data from the MOBILE trial stratified according to underlying glycemic control metrics at baseline. We hypothesized that participants with worse control at baseline (by HbA1c and CGM time-in-range 70–180 mg/dL [TIR] categories) would have the greatest benefit from adding CGM to their diabetes care over 8 months of follow-up.

## Methods

The MOBILE trial was a multicenter, randomized, open-label parallel-group trial conducted at 15 centers in the United States. The protocol and informed consent form were approved by a central institutional review board for 14 centers and a local board for 1 center. Details of the protocol and methods have been previously published^21^; relevant aspects of the protocol are summarized below.

### Study participants and trial design

As previously reported, the MOBILE trial included 175 adults with type 2 diabetes using basal insulin, with 116 randomly assigned to the CGM group and 59 to the BGM group. Mean age was 57 years (range 33 to 79); 50% were female. The race/ethnicity distribution was 47% white, 28% Hispanic, 18% black, 5% Asian, and 2% other. Mean (±standard deviation [SD]) baseline HbA1c was 9.1% ± 0.9% and mean (±SD) baseline TIR 70–180 mg/dL was 40% ± 25%.^21,22^ Potential participants were recruited from primary care practices and were not under the care of an endocrinologist to manage their diabetes. Participants could not have been using prandial insulin at the time of enrollment.

After enrollment, a blinded CGM was worn for up to 10 days before randomization to collect baseline CGM data and participants must have provided at least 168 h (7 days) of CGM data to be eligible. Blood was drawn before randomization to measure HbA1c.

Participants were randomly assigned to the CGM or BGM groups in a 2:1 ratio. The CGM group was provided with a Dexcom™ G6 continuous glucose monitor and BGM and instructed to use the CGM continuously. The BGM group were provided a BGM and asked to perform BGM fasting and postprandial testing one to three times daily. Participants in the BGM group wore a blinded CGM in the 10 days after the 3-month follow-up clinic visit and 10 days leading up to the 8-month follow-up visit.

To obtain a comparable sample in the CGM group, data collected in the 10 days after month 3 and 10 days before month 8 were used to compute CGM outcomes. CGM metrics were calculated by pooling data from the 3- and 8-month CGM wear periods. HbA1c was measured by a central laboratory and collected at randomization, month 3, and 8. Changes in diabetes medications were made by the primary care provider.

### Statistical methods

Participants were divided into joint and mutually exclusive subgroups based on their baseline HbA1c and baseline TIR. The joint subgroups were created to increase sample size when comparing treatment arms within each subgroup, while mutually exclusive groups were used to evaluate interactions. The primary outcomes for this analysis were change in TIR and change in HbA1c from baseline to follow-up. Additional outcomes included change in mean glucose, change in time above 180, 250, and 300 mg/dL, change in total daily insulin, adding or stopping diabetes medication, adding prandial insulin, and one or more hyperglycemic events defined as at least 90 min >300 mg/dL in a 120-min window. Treatment group comparisons were performed within each joint subgroup, and interactions between treatment group and baseline HbA1c and baseline TIR mutually exclusive groups were tested.

A longitudinal mixed effects linear model adjusting for baseline value and clinical site as a random effect was used to compare means of continuous outcomes between treatment groups. The models included outcomes at baseline and pooled month 8 in the response. The 95% confidence interval for the mean treatment group differences is reported for each continuous outcome. An interaction term for the continuous version of the subgroup factor by treatment was added to the models to test for interactions. Logistic regression models with baseline value and a random clinical site effect were used to compare binary outcomes between groups. Adjusted risk differences for the binary outcomes were calculated as in Kleinman and Norton^23^ and confidence intervals were calculated using a bootstrap. For binary outcomes tested within subgroups with a small sample size or few events, Barnard's exact test was used instead.

All *P*-values and confidence intervals reported are two-sided. For this post hoc analysis, no adjustments were made for multiple comparisons and results are considered exploratory. Analyses were conducted using SAS version 9.4 (SAS Institute, Cary, NC).

## Results

### Subgroups based on baseline HbA1c

**FIG. 1.**
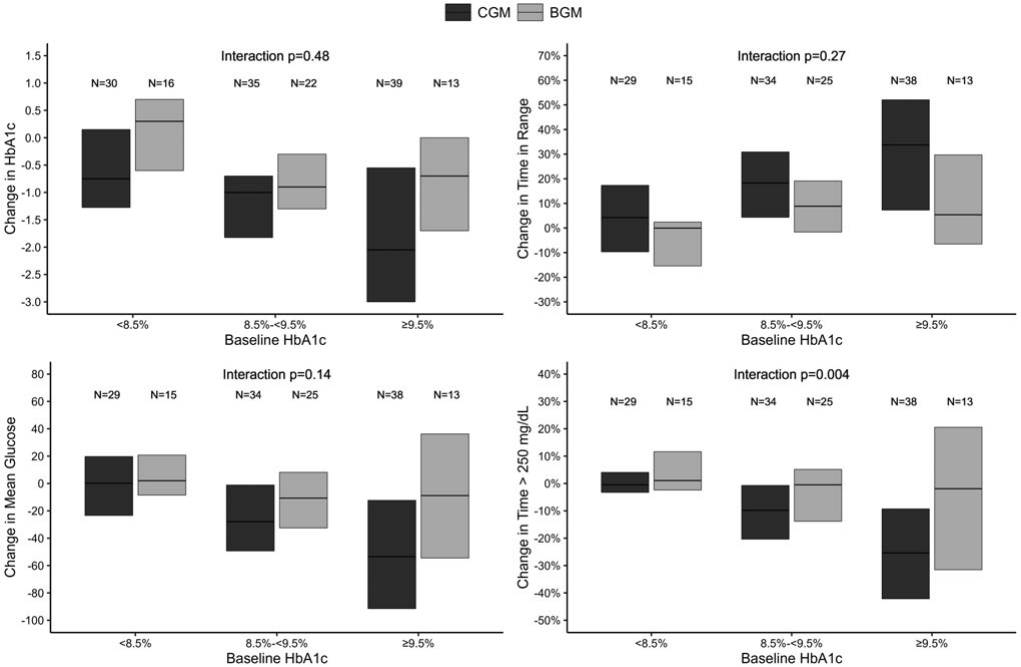
Change in HbA1c, TIR, mean glucose, and time above 250 mg/dL by baseline HbA1c. HbA1c, hemoglobin A1c.

In the overall analysis, TIR increased from 40% at baseline to 58% at 8 months with CGM and from 40% to 45% with BGM (Table 1). In subgroups based on baseline HbA1c, compared with the BGM Group, the CGM Group had 14% (3.4 h per day) greater increase in TIR for participants with baseline HbA1c ≥8.5%, 14% (3.4 h per day) greater increase for baseline HbA1c ≥9.0%, 22% (5.3 h per day) greater increase for baseline HbA1c ≥9.5%, and 32% (7.7 h per day) greater increase for baseline HbA1c ≥10.0% (Table 2). Despite this trend toward greater improvement with higher baseline HbA1c, the *P*-value for interaction was 0.27 when evaluating whether the treatment group difference in TIR varied according to higher baseline HbA1c (Fig. 1). A greater improvement in mean glucose also was seen for subgroups with higher baseline HbA1c, although *P*-value for interaction was 0.14 (Fig. 1).

**Table 1.** Summary of Outcomes by Treatment Group and Baseline Hemoglobin A1c

			Baseline HbA1c							
	Overall		≥8.5%		≥9.0%		≥9.5%		≥10.0%	
	CGM* N* = 116	BGM* N* = 59	CGM* N* = 84	BGM* N* = 41	CGM* N* = 59	BGM* N* = 30	CGM* N* = 44	BGM* N* = 15	CGM* N* = 26	BGM* N* = 9
Baseline TIR 70–180 mg/dL	40% ± 26%	40% ± 25%	32% ± 24%	32% ± 23%	27% ± 23%	26% ± 21%	23% ± 22%	21% ± 15%	22% ± 21%	24% ± 17%
Baseline mean glucose (mg/dL)	209 ± 48	206 ± 45	224 ± 47	220 ± 45	233 ± 47	230 ± 44	242 ± 46	245 ± 39	245 ± 46	234 ± 41
Baseline HbA1c (%)	9.1 ± 1.0	9.0 ± 0.9	9.6 ± 0.8	9.4 ± 0.7	9.9 ± 0.6	9.7 ± 0.6	10.2 ± 0.5	10.2 ± 0.5	10.5 ± 0.4	10.6 ± 0.4
CGM metrics change from baseline	*N* = 102	*N* = 54	*N* = 72	*N* = 38	*N* = 51	*N* = 28	*N* = 38	*N* = 13	*N* = 20	*N* = 8
TIR 70–180 mg/dL	17% ± 28%	5% ± 26%	23% ± 28%	10% ± 26%	24% ± 31%	12% ± 29%	28% ± 33%	9% ± 34%	31% ± 37%	−2% ± 35%
Increase ≥5%	67 (66%)	25 (46%)	53 (74%)	22 (58%)	37 (73%)	17 (61%)	30 (79%)	7 (54%)	16 (80%)	3 (38%)
Increase ≥10%	60 (59%)	20 (37%)	47 (65%)	18 (47%)	34 (67%)	15 (54%)	27 (71%)	6 (46%)	15 (75%)	2 (25%)
Increase ≥15%	54 (53%)	16 (30%)	44 (61%)	14 (37%)	32 (63%)	13 (46%)	25 (66%)	5 (38%)	14 (70%)	2 (25%)
T > 180 mg/dL	−17% ± 28%	−5% ± 26%	−23% ± 28%	−10% ± 27%	−24% ± 31%	−13% ± 30%	−29% ± 33%	−10% ± 35%	−31% ± 37%	2% ± 36%
T > 250 mg/dL	−12% ± 23%	−1% ± 25%	−18% ± 24%	−4% ± 26%	−19% ± 26%	−7% ± 28%	−24% ± 27%	−3% ± 39%	−27% ± 29%	12% ± 41%
T > 300 mg/dL	−6% ± 15%	1% ± 17%	−9% ± 16%	0% ± 18%	−10% ± 18%	−1% ± 20%	−13% ± 18%	1% ± 28%	−16% ± 18%	12% ± 29%
Mean glucose (mg/dL)	−27 ± 47	−4 ± 50	−38 ± 48	−11 ± 52	−40 ± 53	−17 ± 59	−50 ± 54	−11 ± 78	−55 ± 56	20 ± 80
HbA1c change from baseline	*N* = 104	*N* = 51	*N* = 74	*N* = 35	*N* = 53	*N* = 27	*N* = 39	*N* = 13	*N* = 22	*N* = 8
HbA1c (%)	−1.08 ± 1.48	−0.64 ± 1.17	−1.36 ± 1.39	−0.94 ± 1.11	−1.42 ± 1.58	−1.00 ± 1.18	−1.65 ± 1.58	−0.92 ± 1.49	−2.07 ± 1.46	−0.40 ± 1.48
Decrease by ≥0.5%	76 (73%)	33 (65%)	57 (77%)	26 (74%)	37 (70%)	20 (74%)	29 (74%)	9 (69%)	18 (82%)	4 (50%)
Decrease by ≥1.0%	56 (54%)	20 (39%)	47 (64%)	17 (49%)	34 (64%)	14 (52%)	27 (69%)	6 (46%)	18 (82%)	3 (38%)
Insulin metrics change from baseline	*N* = 97	*N* = 48	*N* = 67	*N* = 34	*N* = 47	*N* = 26	*N* = 35	*N* = 13	*N* = 19	*N* = 8
Total daily Insulin (units/kg)	0.02 ± 0.24	0.05 ± 0.22	0.05 ± 0.21	0.08 ± 0.20	0.08 ± 0.24	0.10 ± 0.22	0.07 ± 0.27	0.15 ± 0.17	0.12 ± 0.33	0.15 ± 0.15
HbA1c at month 8	*N* = 105	*N* = 51	*N* = 74	*N* = 35	*N* = 53	*N* = 27	*N* = 39	*N* = 13	*N* = 22	*N* = 8
<7.0%	20 (19%)	5 (10%)	10 (14%)	2 (6%)	5 (9%)	1 (4%)	4 (10%)	1 (8%)	2 (9%)	0 (0%)
<7.5%	40 (38%)	12 (24%)	21 (28%)	7 (20%)	13 (25%)	6 (22%)	8 (21%)	2 (15%)	6 (27%)	0 (0%)
<8.0%	66 (63%)	20 (39%)	44 (59%)	12 (34%)	25 (47%)	8 (30%)	18 (46%)	3 (23%)	11 (50%)	0 (0%)
Medication changes	*N* = 116	*N* = 59	*N* = 84	*N* = 41	*N* = 59	*N* = 30	*N* = 44	*N* = 15	*N* = 26	*N* = 9
Added ≥1 diabetes medication	37 (32%)	24 (41%)	26 (31%)	18 (44%)	19 (32%)	15 (50%)	16 (36%)	8 (53%)	10 (38%)	4 (44%)
Stopped ≥1 diabetes medication	15 (13%)	10 (17%)	10 (12%)	7 (17%)	6 (10%)	5 (17%)	6 (14%)	3 (20%)	3 (12%)	1 (11%)
Added prandial insulin	12 (10%)	9 (15%)	10 (12%)	5 (12%)	9 (15%)	5 (17%)	8 (18%)	3 (20%)	5 (19%)	1 (11%)
Hyperglycemic events at month 8^a^	*N* = 104	*N* = 54	*N* = 74	*N* = 38	*N* = 53	*N* = 28	*N* = 39	*N* = 13	*N* = 21	*N* = 8
≥1 Hyperglycemic event >300 mg/dL	66 (63%)	44 (81%)	49 (66%)	32 (84%)	34 (64%)	25 (89%)	27 (69%)	12 (92%)	13 (62%)	8 (100%)
≥1 Prolonged hyperglycemic event	45 (43%)	31 (57%)	34 (46%)	26 (68%)	26 (49%)	23 (82%)	21 (54%)	12 (92%)	10 (48%)	8 (100%)

^a^ A hyperglycemic event >300 mg/dL is defined as spending a cumulative 90 min or more >300 mg/dL in a 120 min window. A prolonged hyperglycemic event is defined as an event lasting at least 8 h.

BGM, blood glucose meter; CGM, continuous glucose monitoring; HbA1c, hemoglobin A1c; TIR, time-in-range.

**Table 2.** Treatment Group Differences for Outcomes by Baseline Hemoglobin A1c

	Adjusted difference (95% CI) [*P*-value]^a^				
		Baseline HbA1c			
	Overall	≥8.5%	≥9.0%	≥9.5%	≥10.0%
Change from baseline					
TIR 70–180 mg/dL	13% (7 to 20) [<0.001]	14% (5 to 22) [0.001]	14% (3 to 24) [0.01]	22% (7 to 37) [0.005]	32% (11 to 53) [0.004]
Increase ≥5%	21% (9 to 34) [<0.001]	17% (3 to 35) [0.02]	14% (−2 to 35) [0.10]	23% (16 to 34) [<0.001]	39% (17 to 70) [0.001]
Increase ≥10%	23% (13 to 35) [<0.001]	19% (6 to 33) [0.003]	16% (−1 to 34) [0.06]	21% (1 to 42) [0.04]	54% (18 to 76) [0.009]
Increase ≥15%	24% (13 to 36) [<0.001]	25% (9 to 39) [0.004]	17% (−4 to 37) [0.11]	24% (3 to 44) [0.02]	43% (9 to 67) [0.02]
T > 180 mg/dL	−13% (−19 to −6) [<0.001]	−13% (−22 to −5) [0.002]	−13% (−24 to −2) [0.02]	−21% (−37 to −6) [0.009]	−31% (−52 to −10) [0.006]
T > 250 mg/dL	−11% (−15 to −7) [<0.001]	−14% (−19 to −8) [<0.001]	−13% (−20 to −5) [<0.001]	−24% (−34 to −13) [<0.001]	−33% (−46 to −19) [<0.001]
T > 300 mg/dL	−6% (−9 to −4) [<0.001]	−8% (−12 to −5) [<0.001]	−9% (−13 to −4) [<0.001]	−17% (−24 to −9) [<0.001]	−23% (−32 to −15) [<0.001]
Mean glucose (mg/dL)	−22 (−34 to −10) [<0.001]	−24 (−39 to −9) [0.002]	−22 (−42 to −2) [0.03]	−39 (−68 to −11) [0.007]	−60 (−95 to −24) [0.002]
HbA1c (%)	−0.43 (−0.79 to −0.06) [0.02]	−0.37 (−0.81 to 0.07) [0.10]	−0.25 (−0.78 to 0.29) [0.37]	−0.77 (−1.58 to 0.05) [0.06]	−1.52 (−2.55 to −0.50) [0.005]
Decrease by ≥0.5%	10% (−0 to 21) [0.05]	6% (−9 to 27) [0.56]	−4% (−16 to 13) [0.56]	5% (−12 to 24) [0.59]	30% (−1 to 59) [0.06]
Decrease by ≥1.0%	15% (−1 to 31) [0.07]	15% (−7 to 36) [0.18]	9% (−12 to 32) [0.44]	21% (−4 to 46) [0.09]	42% (8 to 68) [0.02]
Total daily insulin (units/kg)	−0.03 (−0.10 to 0.05) [0.51]	−0.03 (−0.11 to 0.06) [0.52]	−0.03 (−0.14 to 0.08) [0.57]	−0.09 (−0.25 to 0.07) [0.25]	−0.05 (−0.30 to 0.21) [0.71]
HbA1c at month 8					
<7.0%^b^	12% (1 to 25) [0.04]	9% (−8 to 25) [0.25]	5% (−14 to 23) [0.45]	−8% (−29 to 10) [0.38]	9% (−25 to 29) [0.45]
<7.5%^b^	17% (0 to 34) [0.05]	10% (−14 to 31) [0.39]	5% (−22 to 29) [0.67]	−3% (−22 to 14) [0.72]	27% (−12 to 50) [0.13]
<8.0%^b^	25% (14 to 36) [<0.001]	27% (10 to 42) [0.001]	19% (−4 to 40) [0.10]	15% (−20 to 47) [0.39]	50% (11 to 69) [0.01]
Medication changes					
Added ≥1 diabetes medication	−9% (−21 to 4) [0.17]	−13% (−31 to 4) [0.14]	−17% (−35 to 2) [0.08]	−14% (−38 to 11) [0.29]	−8% (−52 to 35) [0.74]
Stopped ≥1 diabetes medication	−4% (−15 to 5) [0.39]	−6% (−20 to 6) [0.35]	−9% (−23 to 6) [0.24]	−12% (−32 to 8) [0.23]	−3% (−34 to 20) [0.93]
Added prandial insulin	−5% (−17 to 4) [0.31]	−1% (−15 to 9) [0.93]	−2% (−16 to 9) [0.83]	−3% (−21 to 10) [0.74]	6% (−53 to 40) [0.57]
Hyperglycemic events at month 8^c^					
≥1 Hyperglycemic event >300 mg/dL^b^	−18% (−31 to −6) [0.006]	−17% (−34 to 3) [0.08]	−22% (−40 to 0) [0.05]	−14% (−33 to 8) [0.17]	−38% (−60 to −1) [0.04]
≥1 Prolonged hyperglycemic event^b^	−15% (−25 to −4) [0.005]	−23% (−41 to −2) [0.03]	−32% (−46 to −14) [0.002]	−34% (−53 to −7) [0.02]	−52% (−71 to −11) [0.01]

^a^ For continuous outcomes, estimates, confidence intervals, and *P*-values were calculated from a repeated measures mixed effects linear regression model adjusting for clinical site as a random effect. For binary outcomes, the risk difference, confidence intervals, and *P*-values were estimated from a logistic regression model adjusting for the baseline value as a fixed effect and clinical site as a random effect.

^b^ For the ≥10% subgroup, Barnard's exact test was used to estimate the risk difference and *P*-value due to small sample size. This test cannot adjust for the baseline value or random site effect.

^c^ A hyperglycemic event >300 mg/dL is defined as spending a cumulative 90 min or more >300 mg/dL in a 120 min window. A prolonged hyperglycemic event is defined as an event lasting at least 8 h.

**FIG. 2.**
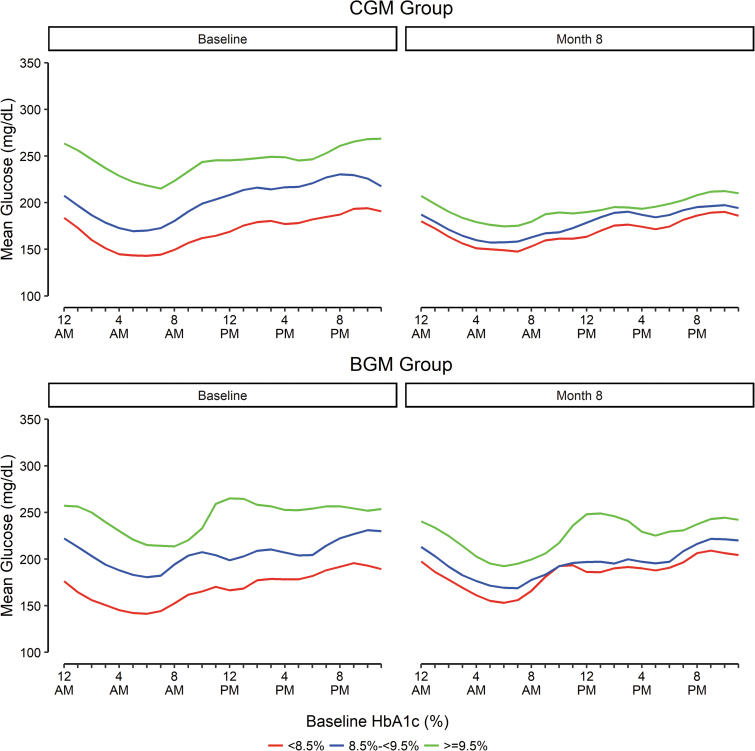
Mean glucose over 24 h by baseline HbA1c. For those with baseline HbA1c <8.5%, mean glucose at baseline was 169 mg/dL in the CGM and BGM groups. For those with baseline HbA1c 8.5% to <9.5%, mean glucose at baseline was 203 and 206 mg/dL in the CGM and BGM groups, respectively. For those with baseline HbA1c ≥9.5%, mean glucose at baseline was 242 and 245 mg/dL in the CGM and BGM groups, respectively. BGM, blood glucose meter; CGM, continuous glucose monitoring. Color images are available online.

The mean CGM glucose concentrations across the 24 h of the day are shown in Figure 2 and highlight the marked improvement from baseline to 8 months during both daytime and nighttime hours in those with baseline HbA1c ≥9.5% using CGM. Similar trends toward greater hyperglycemia reduction with higher baseline HbA1c were observed for hyperglycemia outcomes, particularly for time >250 mg/dL for which the *P*-value for interaction was 0.004 (Fig. 1).

In the overall analysis with HbA1c as the outcome, mean change in HbA1c was −1.08% for the CGM Group and −0.64% for the BGM Group (Table 1). In subgroups based on baseline HbA1c, compared with the BGM Group, the CGM Group had 0.37% greater reduction in HbA1c for participants with baseline HbA1c ≥8.5%, 0.25% greater reduction for baseline HbA1c ≥9.0%, 0.77% greater reduction for baseline HbA1c ≥9.5%, and 1.52% greater reduction for baseline HbA1c ≥10.0% (Table 2).

There were no significant differences between treatment groups for change in total daily insulin, adding prandial insulin, or change in diabetes medications overall and by baseline HbA1c subgroups (Table 2). Mean baseline time <54 mg/dL was 0.05%, 0.04%, 0.04%, and 0.08% for those with baseline HbA1c ≥8.5%, ≥9.0%, ≥9.5%, and ≥10.0%, respectively. There was little change in time <54 mg/dL from baseline to follow-up in the two treatment arms. For the CGM Group, mean (±SD) CGM use over 8 months was 5.7 ± 1.4 days per week for participants with a baseline HbA1c <9.5% and 5.1 ± 1.8 days per week for participants with a baseline HbA1c ≥9.5%.

### Subgroups based on baseline TIR

The change in glycemic outcomes in subgroups based on baseline TIR are presented in Supplementary Table S1 and yielded similar results to the stratified baseline HbA1c analysis. Compared with the BGM Group, the CGM Group had 10% (2.4 h per day) greater increase in TIR for participants with a baseline TIR ≥50% and 17% (4.1 h per day) greater increase for participants with a baseline TIR ≤30% (Supplementary Table S2). Similar trends were observed for change in HbA1c and hyperglycemia outcomes, but no significant interaction was found between treatment group and baseline TIR (Supplementary Fig. S1).

## Discussion

This post hoc analysis of the MOBILE trial demonstrates that among participants with worse glycemic control at baseline, the use of CGM was associated with a substantial increase in TIR and reduction in HbA1c compared with use of BGM alone. The most remarkable benefit from CGM in those with the highest baseline HbA1c values appears to be a reduction in time spent with glucose values exceeding 250 mg/dL.

Limited data from clinical trials and observational studies suggest that CGM may induce a larger treatment effect among patients with higher HbA1c or lower TIR at baseline, although most studies have included patients on intensive insulin therapy. A post hoc analysis of the DIAMOND trial evaluating CGM use in adults with both type 1 and type 2 diabetes on intensive insulin therapy reported the greatest HbA1c improvement in those with an initial HbA1c value ≥9%.^16^ Similar findings were observed among participants with lower baseline TIR.^17^ Our results are consistent with and expand these findings to patients with type 2 diabetes on less intensive insulin therapy. The marked reduction in time spent with glucose >250 mg/dL in the subgroup with baseline HbA1c values ≥10% was achieved without significant differences in diabetes medication changes between groups, including the initiation of prandial insulin.

The use of CGM provides patients not only with continuous glucose data, but also information regarding impending glucose excursions. These real-time data, along with alarms to detect glycemic excursions, have the potential to generate a diverse array of behavioral changes aimed at maintaining glucose values within a desired target range. A prior survey investigating management of type 1 and type 2 diabetes using CGM showed most behavioral responses prompted by CGM data, or alerts were associated with modifications to prandial or corrective insulin dosing or the ability to detect and respond to hypoglycemia.^20,24^

There are limited data on behavior changes associated with glycemic improvement in patients with type 2 diabetes on less intensive insulin regimens. Small studies have shown that modifications in diet, physical activity, and medication adherence motivated by ongoing CGM feedback are important factors in improving glycemic control in this population that may help reduce the need for intensification of treatment regimens.^7,8,11^ The primary results of the MOBILE trial confirmed the utility of CGM with alarms as an important behavior modification tool leading to a clinically meaningful reduction in HbA1c in patients with type 2 diabetes treated with basal insulin therapy.^21^ In this analysis, we observed a large treatment effect among patients with baseline HbA1c values ≥10% for achieving both a greater than 1% reduction in HbA1c (42% adjusted difference vs. BGM, *P* = 0.02) and achieving HbA1c levels <8% (50% adjusted difference vs. BGM, *P* = 0.001).

Importantly, this improvement in glycemic control was not associated with increased rates of hypoglycemia. These data suggest that use of CGM promotes behavioral modifications that can lead to significant reductions in HbA1c in those with very high HbA1c levels at baseline, potentially avoiding the need for intensive insulin therapy.

Limitations of this post hoc analysis include the small sample sizes of the stratified groups leading to low statistical power and imprecise effect estimates. The observed glycemic improvement trend does, however, suggest a causal relationship between the magnitude of treatment effect of CGM according to categories of glycemic control at baseline that is consistent for HbA1c and TIR categories.

## Conclusion

Overall, the findings of this post hoc analysis suggest trends of greater treatment effect on glycemic control outcomes with CGM compared with BGM in those with worse baseline glycemic control, similar to what has been observed with other therapeutic interventions. It is possible that behavior modifications prompted by real-time glucose data can provide significant additive glycemic control benefit among patients with very high initial HbA1c on less intensive insulin regimens. Further research is needed to understand long-term behavioral changes and cost-effectiveness of CGM in real-world settings for improvement in glycemic control in diverse populations of patients with poorly controlled type 2 diabetes.

## Supplementary Material

Supplemental data

Supplemental data

Supplemental data
