# Supplementary material for: Magnitude of Glycemic Improvement in Patients with Type 2 Diabetes Treated with Basal Insulin: Subgroup Analyses from the MOBILE Study
Source: Diabetes Technol Ther. 2022 May 10;24(5):324–31. doi: 10.1089/dia.2021.0489 (PMC9127836; doi:10.1089/dia.2021.0489)
Supplement: Supplemental data [file Supp_TableS1.docx]

## Supplemental Table S1. Summary of Outcomes by Baseline Time in Range

|  |  | | **Baseline Time in Range 70-180 mg/dL** | | | | | | | |
| --- | --- | --- | --- | --- | --- | --- | --- | --- | --- | --- |
|  | **Overall** | | **≤ 30%** | | **≤ 40%** | | **≤ 50%** | | **> 50%** | |
|  | **CGM**  **N=116** | **BGM**  **N=59** | **CGM**  **N=41** | **BGM**  **N=26** | **CGM**  **N=61** | **BGM**  **N=30** | **CGM**  **N=73** | **BGM**  **N=37** | **CGM**  **N=41** | **BGM**  **N=22** |
| Baseline TIR 70-180 mg/dL | 40% ± 26% | 40% ± 25% | 13% ± 10% | 16% ± 9% | 20% ± 14% | 18% ± 11% | 24% ± 15% | 24% ± 15% | 69% ± 11% | 68% ± 10% |
| Baseline HbA1c (%) | 9.1 ± 1.0 | 9.0 ± 0.9 | 9.9 ± 0.8 | 9.5 ± 0.8 | 9.6 ± 0.9 | 9.6 ± 0.8 | 9.5 ± 0.9 | 9.4 ± 0.8 | 8.5 ± 0.7 | 8.5 ± 0.7 |
| **CGM Metrics Change from Baseline ^a^** | N=102 | N=54 | N=35 | N=23 | N=53 | N=27 | N=63 | N=34 | N=39 | N=20 |
| TIR 70-180 mg/dL | 17% ± 28% | 5% ± 26% | 38% ± 24% | 20% ± 22% | 33% ± 24% | 17% ± 24% | 28% ± 26% | 14% ± 25% | 0% ± 22% | -10% ± 20% |
| Increase ≥ 5% | 67 (66%) | 25 (46%) | 32 (91%) | 16 (70%) | 46 (87%) | 17 (63%) | 52 (83%) | 21 (62%) | 15 (38%) | 4 (20%) |
| Increase ≥ 10% | 60 (59%) | 20 (37%) | 30 (86%) | 14 (61%) | 42 (79%) | 15 (56%) | 46 (73%) | 18 (53%) | 14 (36%) | 2 (10%) |
| Increase ≥ 15% | 54 (53%) | 16 (30%) | 28 (80%) | 13 (57%) | 40 (75%) | 14 (52%) | 44 (70%) | 16 (47%) | 10 (26%) | 0 (0%) |
| T > 180 mg/dL | -17% ± 28% | -5% ± 26% | -38% ± 24% | -21% ± 23% | -33% ± 24% | -17% ± 25% | -28% ± 26% | -14% ± 25% | 1% ± 22% | 10% ± 20% |
| T > 250 mg/dL | -12% ± 23% | -1% ± 25% | -33% ± 23% | -13% ± 25% | -26% ± 22% | -9% ± 27% | -22% ± 23% | -5% ± 28% | 3% ± 12% | 8% ± 15% |
| T > 300 mg/dL | -6% ± 15% | 1% ± 17% | -18% ± 18% | -5% ± 19% | -13% ± 17% | -2% ± 20% | -11% ± 16% | 0% ± 21% | 2% ± 7% | 3% ± 8% |
| Mean Glucose (mg/dL) | -27 ± 47 | -4 ± 50 | -66 ± 43 | -29 ± 50 | -54 ± 42 | -21 ± 55 | -46 ± 45 | -16 ± 56 | 3 ± 33 | 15 ± 32 |
| **HbA1c Change from Baseline** | N=104 | N=51 | N=36 | N=20 | N=54 | N=24 | N=64 | N=31 | N=38 | N=20 |
| HbA1c (%) | -1.08 ± 1.48 | -0.64 ± 1.17 | -1.59 ± 1.53 | -0.99 ± 1.24 | -1.38 ± 1.42 | -0.92 ± 1.24 | -1.27 ± 1.43 | -0.81 ± 1.25 | -0.78 ± 1.50 | -0.37 ± 1.00 |
| Decrease by ≥ 0.5% | 76 (73%) | 33 (65%) | 28 (78%) | 14 (70%) | 41 (76%) | 16 (67%) | 47 (73%) | 21 (68%) | 28 (74%) | 12 (60%) |
| Decrease by ≥ 1.0% | 56 (54%) | 20 (39%) | 25 (69%) | 11 (55%) | 33 (61%) | 12 (50%) | 37 (58%) | 15 (48%) | 18 (47%) | 5 (25%) |
| **Insulin Metrics Change from Baseline** | N=97 | N=48 | N=32 | N=18 | N=50 | N=22 | N=60 | N=29 | N=35 | N=19 |
| Total Daily Insulin (units) | 0.02 ± 0.24 | 0.05 ± 0.22 | 0.08 ± 0.26 | 0.17 ± 0.17 | 0.03 ± 0.26 | 0.13 ±0.18 | 0.03 ± 0.24 | 0.12 ± 0.21 | -0.01 ± 0.22 | -0.07 ± 0.18 |
| **HbA1c at Month 8 ^b^** | N=105 | N=51 | N=36 | N=20 | N=55 | N=24 | N=65 | N=31 | N=38 | N=20 |
| < 7.0% | 20 (19%) | 5 (10%) | 4 (11%) | 2 (10%) | 6 (11%) | 2 (8%) | 6 (9%) | 3 (10%) | 14 (37%) | 2 (10%) |
| < 7.5% | 40 (38%) | 12 (24%) | 12 (33%) | 4 (20%) | 19 (35%) | 5 (21%) | 20 (31%) | 7 (23%) | 20 (53%) | 5 (25%) |
| < 8.0% | 66 (63%) | 20 (39%) | 19 (53%) | 6 (30%) | 33 (60%) | 7 (29%) | 39 (60%) | 10 (32%) | 26 (68%) | 10 (50%) |
| **Medication Changes** | N=116 | N=59 | N=41 | N=26 | N=61 | N=30 | N=73 | N=37 | N=41 | N=22 |
| Added ≥ 1 Diabetes Medication | 37 (32%) | 24 (41%) | 19 (46%) | 11 (42%) | 25 (41%) | 12 (40%) | 28 (38%) | 18 (49%) | 9 (22%) | 6 (27%) |
| Stopped ≥ 1 Diabetes Medication | 15 (13%) | 10 (17%) | 5 (12%) | 2 (8%) | 9 (15%) | 2 (7%) | 10 (14%) | 7 (19%) | 5 (12%) | 3 (14%) |
| Added Prandial Insulin | 12 (10%) | 9 (15%) | 8 (20%) | 4 (15%) | 10 (16%) | 4 (13%) | 11 (15%) | 7 (19%) | 1 (2%) | 2 (9%) |
| **Hyperglycemic Events at Month 8 ^a^** | N=104 | N=54 | N=35 | N=23 | N=53 | N=27 | N=63 | N=34 | N=39 | N=20 |
| ≥ 1 Hyperglycemic Event > 300 mg/dL | 66 (63%) | 44 (81%) | 25 (71%) | 22 (96%) | 36 (68%) | 25 (93%) | 44 (70%) | 31 (91%) | 21 (54%) | 13 (65%) |
| ≥ 1 Prolonged Hyperglycemic Event | 45 (43%) | 31 (57%) | 17 (49%) | 19 (83%) | 25 (47%) | 22 (81%) | 31 (49%) | 24 (71%) | 13 (33%) | 7 (35%) |

^a^ A hyperglycemic event > 300 mg/dL is defined as spending a cumulative 90 minutes or more > 300 mg/dL in a 120 minute window. A prolonged hyperglycemic event is defined as an event lasting at least 8 hours.
