# Supplementary material for: Magnitude of Glycemic Improvement in Patients with Type 2 Diabetes Treated with Basal Insulin: Subgroup Analyses from the MOBILE Study
Source: Diabetes Technol Ther. 2022 May 10;24(5):324–31. doi: 10.1089/dia.2021.0489 (PMC9127836; doi:10.1089/dia.2021.0489)
Supplement: Supplemental data [file Supp_TableS2.docx]

|  | **Adjusted Difference (95% CI) [P-Value] ^a^** | | | | |
| --- | --- | --- | --- | --- | --- |
|  |  | **Baseline Time in Range 70-180 mg/dL** | | | |
|  | **Overall** | **≤ 30%** | **≤ 40%** | **≤ 50%** | **> 50%** |
| **Change from Baseline** |  |  |  |  |  |
| TIR 70-180 mg/dL | 13% (7%, 20%) [<0.001] | 17% (5%, 29%) [0.008] | 18% (7%, 28%) [0.001] | 15% (5%, 24%) [0.002] | 10% (-1%, 21%) [0.08] |
| Increase ≥ 5% | 21% (9%, 34%) [<0.001] | 22% (9%, 37%) [<0.001] | 25% (11%, 41%) [<0.001] | 22% (5%, 42%) [0.007] | 17% (-8%, 43%) [0.20] |
| Increase ≥ 10% | 23% (13%, 35%) [<0.001] | 23% (10%, 39%) [<0.001] | 26% (12%, 39%) [<0.001] | 22% (11%, 32%) [<0.001] | 23% (-4%, 50%) [0.09] |
| Increase ≥ 15% ^b^ | 24% (13%, 36%) [<0.001] | 21% (4%, 39%) [0.02] | 23% (3%, 41%) [0.02] | 24% (8%, 37%) [0.004] | 26% (7%, 41%) [0.01] |
| T > 180 mg/dL | -13% (-19%, -6%) [<0.001] | -16% (-29%, -4%) [0.01] | -18% (-28%, -7%) [0.001] | -14% (-24%, -5%) [0.003] | -9% (-20%, 2%) [0.12] |
| T > 250 mg/dL | -11% (-15%, -7%) [<0.001] | -15% (-24%, -6%) [0.001] | -18% (-25%, -10%) [<0.001] | -16% (-23%, -10%) [<0.001] | -3% (-8%, 1%) [0.16] |
| T > 300 mg/dL | -6% (-9%, -4%) [<0.001] | -8% (-14%, -2%) [0.009] | -11% (-15%, -6%) [<0.001] | -10% (-14%, -6%) [<0.001] | 0% (-2%, 2%) [0.90] |
| Mean Glucose (mg/dL) | -22 (-34, -10) [<0.001] | -28 (-52, -5) [0.02] | -30 (-49, -10) [0.003] | -27 (-45, -10) [0.003] | -11 (-28, 7) [0.24] |
| HbA1c (%) | -0.43 (-0.79, -0.06) [0.02] | -0.40 (-1.05, 0.24) [0.22] | -0.55 (-1.11, 0.02) [0.06] | -0.45 (-0.93, 0.04) [0.07] | -0.39 (-0.97, 0.19) [0.19] |
| Decrease by ≥ 0.5% | 10% (-0%, 21%) [0.05] | 8% (-7%, 30%) [0.39] | 11% (-5%, 33%) [0.22] | 8% (-8%, 29%) [0.40] | 10% (-13%, 33%) [0.40] |
| Decrease by ≥ 1.0% | 15% (-1%, 31%) [0.07] | 12% (-5%, 32%) [0.19] | 15% (-5%, 34%) [0.14] | 11% (-6%, 28%) [0.24] | 19% (-8%, 47%) [0.17] |
| Total Daily Insulin (units) | -0.03 (-0.10, 0.05) [0.51] | -0.08 (-0.22, 0.06) [0.24] | -0.08 (-0.20, 0.03) [0.15] | -0.10 (-0.20, -0.00) [0.05] | 0.06 (-0.06, 0.17) [0.31] |
| **HbA1c at Month 8** |  |  |  |  |  |
| < 7.0% | 12% (1%, 25%) [0.04] | 5% (-9%, 20%) [0.41] | 1% (-14%, 16%) [0.86] | -2% (-14%, 10%) [0.66] | 24% (8%, 42%) [0.004] |
| < 7.5% | 17% (0%, 34%) [0.05] | 21% (-0%, 42%) [0.05] | 13% (-12%, 38%) [0.29] | 12% (-8%, 31%) [0.23] | 23% (-1%, 45%) [0.06] |
| < 8.0% | 25% (14%, 36%) [<0.001] | 29% (11%, 47%) [0.002] | 29% (8%, 50%) [0.008] | 29% (11%, 45%) [0.001] | 17% (-5%, 36%) [0.12] |
| **Medication Changes** |  |  |  |  |  |
| Added ≥ 1 Diabetes Medication | -9% (-21%, 4%) [0.17] | 4% (-14%, 21%) [0.68] | 1% (-18%, 19%) [0.91] | -10% (-29%, 9%) [0.32] | -5% (-27%, 15%) [0.61] |
| Stopped ≥ 1 Diabetes Medication | -4% (-15%, 5%) [0.39] | 4% (-15%, 20%) [0.61] | 7% (-9%, 19%) [0.28] | -5% (-21%, 8%) [0.45] | 2% (-22%, 21%) [0.79] |
| Added Prandial Insulin | -5% (-17%, 4%) [0.31] | 4% (-10%, 16%) [0.55] | 1% (-12%, 13%) [0.88] | -6% (-20%, 7%) [0.37] | -6% (-23%, 8%) [0.27] |
| **Hyperglycemic Events at Month 8 ^c^** |  |  |  |  |  |
| ≥ 1 Hyperglycemic Event > 300 mg/dL | -18% (-31%, -6%) [0.006] | -25% (-46%, -5%) [0.02] | -21% (-43%, 1%) [0.06] | -21% (-38%, -5%) [0.01] | -13% (-33%, 9%) [0.23] |
| ≥ 1 Prolonged Hyperglycemic Event | -15% (-25%, -4%) [0.005] | -33% (-51%, -10%) [0.007] | -31% (-52%, -7%) [0.01] | -21% (-33%, -8%) [0.002] | -1% (-27%, 28%) [0.90] |

## Supplemental Table S2. Treatment Effects for Outcomes by Baseline Time in Range

^a^ For continuous outcomes, estimates, confidence intervals and p-values were calculated from a repeated measures mixed effects linear regression model adjusting for clinical site as a random effect. For binary outcomes the risk difference, confidence intervals and p-values were estimated from a logistic regression model adjusting for the baseline value as a fixed effect and clinical site as a random effect.

^b^ For the >50% sub-group, Barnard’s exact test was used to estimate the risk difference and p-value due to the small sample size. This test cannot adjust for the baseline value or random site effect.

^c^ A hyperglycemic event > 300 mg/dL is defined as spending a cumulative 90 minutes or more > 300 mg/dL in a 120 minute window. A prolonged hyperglycemic event is defined as an event lasting at least 8 hours.
