# Supplementary material for: Magnitude of Glycemic Improvement in Patients with Type 2 Diabetes Treated with Basal Insulin: Subgroup Analyses from the MOBILE Study
Source: Diabetes Technol Ther. 2022 May 10;24(5):324–31. doi: 10.1089/dia.2021.0489 (PMC9127836; doi:10.1089/dia.2021.0489)

## Supplemental Figure S1. Change in HbA1c, Time in Range, Mean Glucose, and Time above 250 mg/dL by Baseline Time in Range


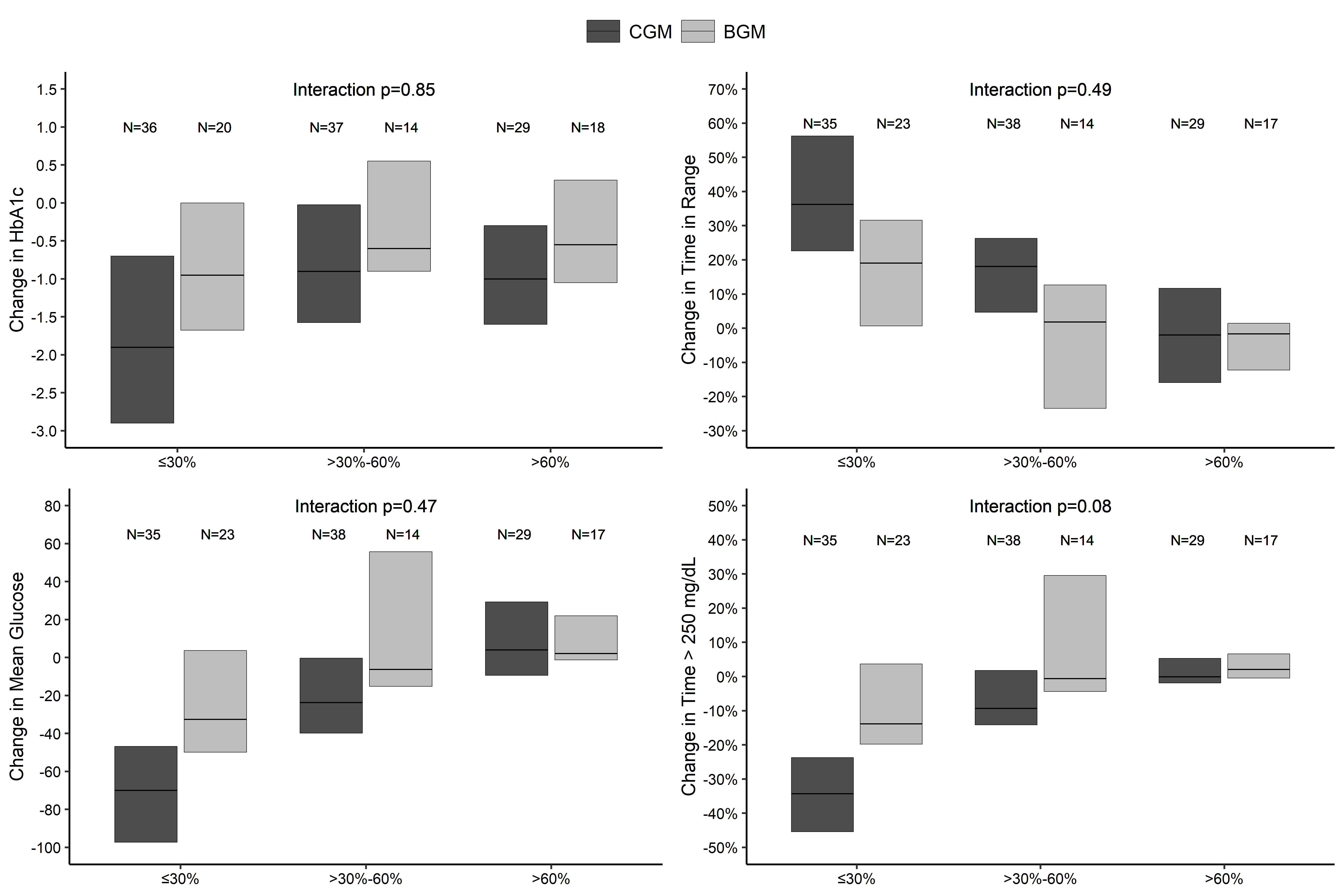

Supplement: Supplemental data [file Supp_FigS1.docx]
